# Rumen Epithelial Transcriptome Analysis Revealed the Regulatory Mechanism of Host Adaptation to Nitrogen Source Replacement Diet in Simmental Cattle

**DOI:** 10.1002/fsn3.71041

**Published:** 2025-09-28

**Authors:** Yushan Li, Xinyi Li, Xinxin Sui, Li Liu, Quratulain Hanif, Fuwen Wang, Li Luo, Ye Bu, Shanshan Xu, Yue Wang, Chuzhao Lei, Fang Sun

**Affiliations:** ^1^ Key Laboratory of Animal Genetics, Breeding and Reproduction of Shaanxi Province, College of Animal Science and Technology Northwest A&F University Xianyang China; ^2^ Key Laboratory of Combining Farming and Animal Husbandry of Ministry of Agriculture, Institute of Animal Husbandry Heilongjiang Academy of Agricultural Sciences Harbin China; ^3^ Department of Life Sciences, School of Science University of Management and Technology Lahore Pakistan; ^4^ Altai Region Livestock Workstation Xinjiang China

**Keywords:** gelatinized urea, regulatory mechanisms, RNA‐seq, rumen epithelial, soybean

## Abstract

Soybean meal, a high‐quality protein in ruminant diets, has been explored by farmers and researchers to replace soybean meal with other nitrogen sources to reduce feed costs due to its high price and limited resources. Gelatinized urea is a commonly used substitute for nitrogen source. In this study, RNA sequencing was performed on rumen epithelial tissues of Simmental beef cattle fed the diet that gelatinized urea replace 10% soybean (experimental group, *n* = 6) as well as Simmental beef cows fed soybean meal normally (control group, *n* = 4) to explore possible regulatory mechanisms in rumen tissues after nitrogen source substitution. The results showed that after the nitrogen source was replaced, the rumen epithelium may increase the nutrient absorption capacity by decreasing the degree of intercellular connectivity, increasing intercellular permeability, and possibly changing the rumen morphology, and the expression of genes related to lipoprotein synthesis was increased in the rumen epithelial tissues, which suggested that the rumen epithelium may have produced a compensatory mechanism to adapt to the changes of the nutrients in the diets.

## Graphical abstract

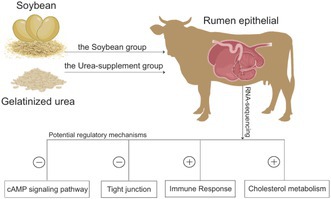

## 1 Introduction

Protein is an essential nutrient for maintaining animal growth and development, however, on the Earth, Crude Protein (CP) and Crude Protein Nitrogen (CPN) are relatively limited in natural abundance, which are insufficient to support the unlimited demand for animal husbandry and the sustainable growth of a healthy global population. Meanwhile, although Non‐Protein Nitrogen (NPN) is relatively abundant in nature, it cannot be directly utilized by humans and non‐ruminants. Therefore, utilizing NPN resources efficiently, converting them into high‐quality protein through ruminants, and using them for human ultimately has become a major challenge for agriculture and animal husbandry (Zhao et al. 2020).

Soybean meal is recognized as a high‐quality protein feed, rich in a variety of essential amino acids, with a balanced and easy profile, providing animals with a directly available source of amino acids. For animals, this protein is essential for maintaining health, strengthening immunity and improving production performance during their growth and metabolism (Wang et al. 2021). The great nutritional characteristics of soybean meal make it an ideal protein source for ruminants such as beef and dairy cattle, especially in Europe, where imported soybean meal is widely used due to its rich content of Rumen Undegradable Protein (RUP) (Reddy et al. 2019).

However, with the improvement of socio‐economic level, the demand for animal products such as meat and dairy products has a marked increase, which has driven the rapid expansion of livestock and poultry farming. At the same time, the shortage of plant‐derived protein feeds, such as soybean, and the persistent rise in prices have led to a significant increase in ruminant feeding costs (Shuhui and Xuefeng 2023). In the face of these challenges, exploring alternative feeds to traditional proteins to reduce the dependence on soybean meal has become a hot topic in modern animal husbandry research (Wei et al. 2024).

Non‐protein nitrogen (NPN) resources, such as urea, are considered as economical solutions for replacing traditional protein feeds due to their abundance in nature and low prices. Ruminants (e.g., cattle, sheep, etc.) have a unique rumen microbial system that can support microbial protein synthesis by directly converting NPNs such as urea to ammonia (NH_3_), which serves as a low‐cost source of nitrogen combines with carbohydrates in feed to synthesize microbial protein (Salami et al. 2021). These microbial proteins are further digested in the animal's rumen to provide necessary amino acids to maintain its normal growth and productive performance.

Gelatinized urea is a protein replacement feed that integrates urea slow release and nitrogen synchronization. The slow release of urea is mainly achieved through the encapsulation of urea by modified starch after pasting, and the reaction between the glyoxal group of starch and the amino group of urea to form a complex, meanwhile, starch can be degraded more easily in the rumen after pasting, which can increase the rate of supplying energy for microorganisms to synthesize bacterial proteins, and promote the synthesis of microbial proteins (Colenbrander et al. 1967; Stiles et al. 1970). Recently, many researchers have combined urea with carbohydrates to achieve synchronized nitrogen release and have developed a variety of products ranging from liquid urea supplements to gelatinized urea (Lewis and McDonald 1958; Johnson 1976). As a cheap and efficient non‐protein nitrogen feed, gelatinized urea is widely used in ruminant feeding around the world, and this feeding strategy can not only alleviate the dilemma caused by the shortage of plant protein feeds, but also improves the efficiency of agricultural resource utilization (Fan et al. 2024).

Although non‐protein nitrogen (NPN) feeds such as urea show many advantages in ruminant feeding, there are certain risks and management challenges associated with their use (Xi and Zhang 2010). Excessive intake of NPN can lead to a sharp increase in ammonia concentration in the rumen, and the excess ammonia will be absorbed into the bloodstream, a process that increases the metabolic burden of the animal, and may cause rumen acidosis or ammonia toxicity if it is in excess. Therefore, in order to better utilize non‐protein nitrogen, such as urea, in the feeding of ruminants, it is very important to study the body's response to such feed after feeding. In this study, we investigated the transcriptomic responses of the rumen epithelium to the replacement of soybean meal with gelatinized urea in Simmental cattle, aiming to uncover the molecular mechanisms underlying host adaptation to an abrupt change in nitrogen source. Based on previous studies, we hypothesized that this dietary shift would trigger coordinated regulatory changes, including the upregulation of lipid metabolism and immune pathways, as well as modulation of barrier tightness, to maintain rumen function and overall homeostasis.

## 2 Materials and Methods

### 2.1 Animal Care and Use and Rumen Tissue Sampling

A set of 10 cattle were divided into two groups: (1) the urea‐supplement group (*n* = 6), which consisted of 4.8% soybeans, 6.6% DDGS, 8.1% corn germ meal, 6.93% rice bran meal, and 0.75% gelatinized urea, While, (2) the soybean group (*n* = 4) consisted of 10.2% soybeans, 6% DDGS, and 6% corn chaff meal, and did not contain urea. The proportion of other nutrients in the dietary formulations was constant for both groups (Table S5). Here, the soyabean group was also used as a control group.

The entire experimental period lasted 124 days, consisting of a 15‐day pre‐trial phase followed by a 109‐day formal trial phase. All experimental cattle were raised in a free‐range system, with each animal provided 14 square meters of activity space, along with free access to water and feed. Feeding was conducted twice daily, at 07:00 in the morning and 16:00 in the afternoon. A feed intake measurement system (Beijing Huiqi Tongda IoT Technology) was used to record daily feed consumption (Table S6).

After slaughtering, a 4‐cm^2^ piece of rumen tissue was obtained from the central region of the ventral sac and rinsed with sterilized PBS buffer (pH = 6.8) before being placed in a 50 mL tube containing RNA later solution (Invitrogen, Carlsbad, CA). The samples were then stored at −80°C until further processing.

### 2.2 RNA Sequencing

Total RNA was extracted using the Trizol (Invitrogen, CA, USA), RNA purity and integrity was monitored by NanoDrop 2000 spectrophotometer (NanoDrop Technologies, Wilmington, DE, USA) and a Bioanalyzer 2100 system (Agilent Technologies, CA, USA). RNA contamination was assessed by 1.5% agarose gel.

The mRNA was purified from the total RNA using poly‐T oligo‐attached magnetic beads. Sequencing libraries were generated from the purified mRNA using the VAHTS Universal V6 RNA‐seq Library Kit for MGI (V azyme, Nanjing, China) following the manufacturer's recommendations with unique index codes. The Library quantification and size was assessed using Qubit 3.0 Fluorometer (Life Technologies, Carlsbad, CA, USA) and Bioanalyzer 2100 system (Agilent Technologies, CA, USA). Subsequently, sequencing was performed on MGI‐SEQ 2000 platform by Frasergen Bioinformatics Co. Ltd. (Wuhan, China).

### 2.3 Rumen Fermentation Measurement

Rumen contents were filtered through four layers of gauze, and pH was immediately measured using a Sartorius‐20 pH meter. Two 10 mL aliquots of rumen fluid were each mixed with 2 mL of 25% metaphosphoric acid and stored at −20°C for later analysis of volatile fatty acids (VFA). VFA concentrations were determined using a gas chromatograph (Shimadzu GC‐2010, Kyoto, Japan).

Data were initially processed using Excel 2021. For normally distributed data, independent samples *t*‐tests were performed using SPSS 23.0; non‐normally distributed data were analyzed using the Wilcoxon rank‐sum test. Group differences were compared using these tests, with results presented as mean ± standard error of the mean (SEM). Statistical significance was set at *p* < 0.05, and *p* < 0.01 was considered highly significant.

### 2.4 Processing RNA‐Seq Data

Paired end FASTQ files were retrieved from the sequencer (with an average read length of 150 bp) which were subjected to quality control using Trimmomatic v.0.39 (Bolger et al. 2014) in PE mode to remove low‐quality reads and adapter sequences contamination. After quality control, the filtered high‐quality clean reads were aligned to the *Bos taurus* reference genome (ARS‐UCD1.2) using STAR v.2.7.11 in a two‐pass alignment mode, generating BAM files for subsequent analysis.

### 2.5 Assembling of Sample Transcripts

StringTie v2.2.1 (Pertea et al. 2015) was used to calculate the expression levels of genes and transcripts from the generated BAM files, producing transcript annotation files and abundance tables for each sample. The same tool was employed to merge the transcripts, harvesting a unified annotation file which was later used to identify the novel transcripts. To expedite assembly, use the annotation file from the ARS‐UCD1.2 reference genome to assist in identifying novel transcripts.

### 2.6 Correlation Analysis of Sample Expression Patterns

The expression levels of the newly assembled transcripts from the BAM files were recalculated. Python scripts were employed to extract the gene and transcript count matrices, as well as the TPM matrix, for subsequent differential expression and clustering analyses.

The Ballgown‐compatible output files were generated using StringTie v2.2.1 and the distance matrix was calculated using the method of Spearman. Lastly, a correlation heatmap and clustering dendrogram was plotted for visualization.

### 2.7 Differential Gene Expression and Principal Component Analysis

DESeq2 (Love et al. 2014) as used for differential expression significance analysis, and the screening threshold was *p*‐adjusted < 0.05, log_2_FC (fold change) > 1 or < −1.

DESeq2 was also used for Principal Component Analysis (PCA) to cluster samples based on normalized gene expression data. Firstly, the counts of gene were normalized based on library size and regularized log transformed. After removing the constant expression of the genes, all genes were compared within the samples, after removing constant gene expressions, to elucidate the principal components in 2D‐cluster visualization.

### 2.8 KEGG and GO Enrichment Analysis

We used Database for Annotation, Visualization, and Integrated Discovery (DAVID) (Huang, Sherman, and Lempicking 2009; Huang, Sherman, Lempicking, et al. 2009) as well as clusterProfiler for gene annotation and functional enrichment analysis. Here, the gene symbols were converted to Entrez IDs by using function for use in subsequent analysis. For functional annotation, we used DAVID was used to analyze the differentially expressed genes (DEGs) in the Kyoto Encyclopedia of Genes and Genomes (KEGG) (Kanehisa and Goto 2000; Minoru et al. 2014) pathway and enrichment according to Gene Ontology (GO) annotation. Moreover, KEGG and GO enrichment were accomplished with the enrichKEGG and enrichGO functions. The Benjamini‐Hochberg (B‐H) correction method was applied on the data, whereby a biological pathway or enrichment was considered significant if the *p*‐adjusted < 0.05.

### 2.9 Differential Gene Set Enrichment Analysis (GSEA)

We performed GSEA with the software provided by the Massachusetts Institute of Technology (Aravind et al. 2005). Normalized enrichment score (NES) and *p*‐adjusted values were used to quantify enrichment magnitude and statistical significance, respectively.

## 3 Results

### 3.1 Identification of the Rumen Epithelial Transcriptome

**FIGURE 1:**
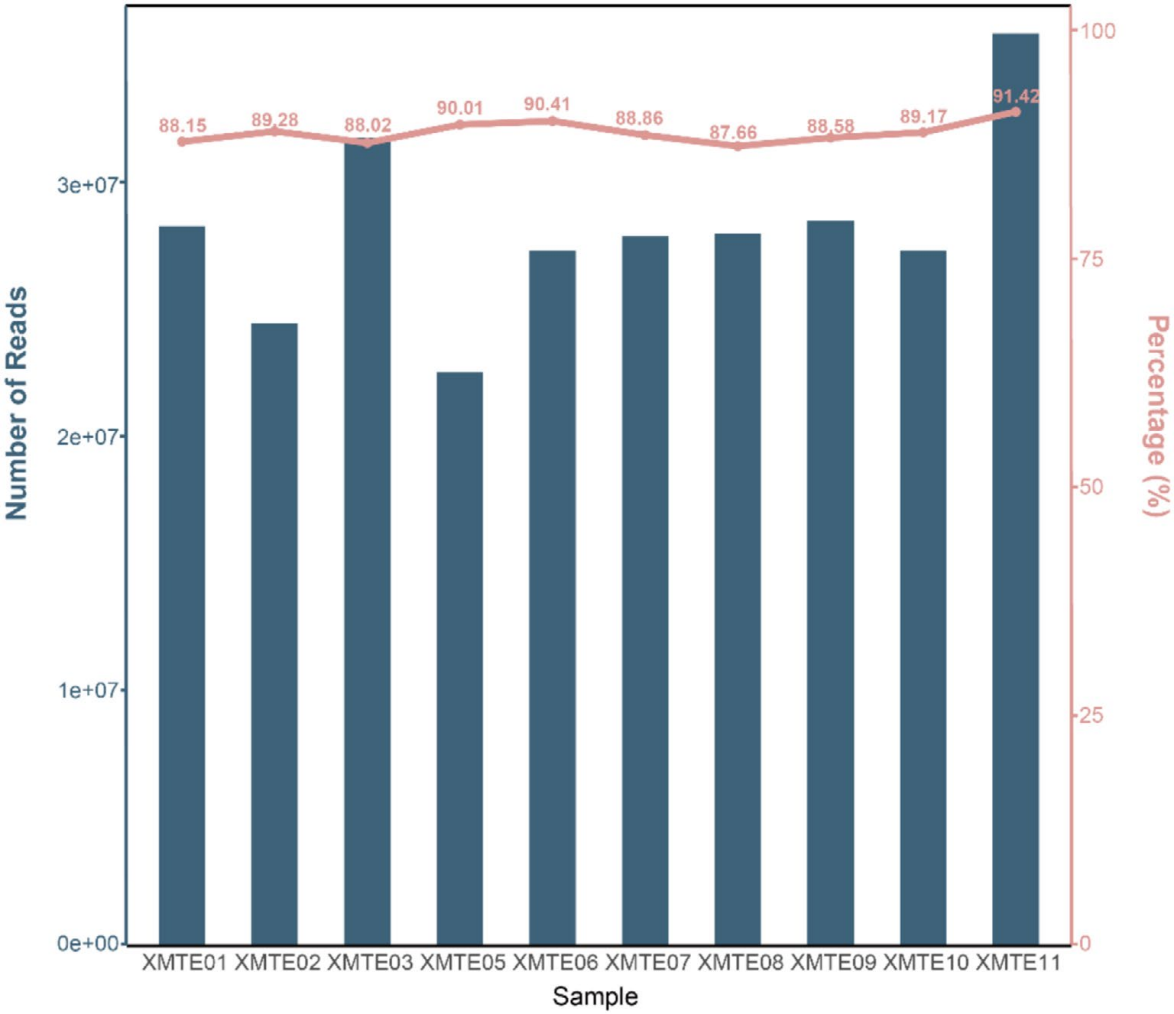
Double *Y*‐axis plot showing the number of reads and comparison rate for each sample in the RNA‐seq after comparison. The *Y*‐axis on the left represents the read length of the samples, and the pink *Y*‐axis on the right represents the comparison rate with the reference genome of cattle.

The urea supplemented and soybean supplemented groups were subjected to transcriptomics yielding an average 28.38 M high‐quality, paired reads per sample with an overall average comparison rate of 89% to the ARS‐UCD1.2 reference genome (Figure 1). A total number of 2407 genes were expressed in rumen epithelial cells (genes with at least one read in at least one sample).

### 3.2 Comparison of Transcription Profiles Between the Two Feeding Groups

**FIGURE 2:**
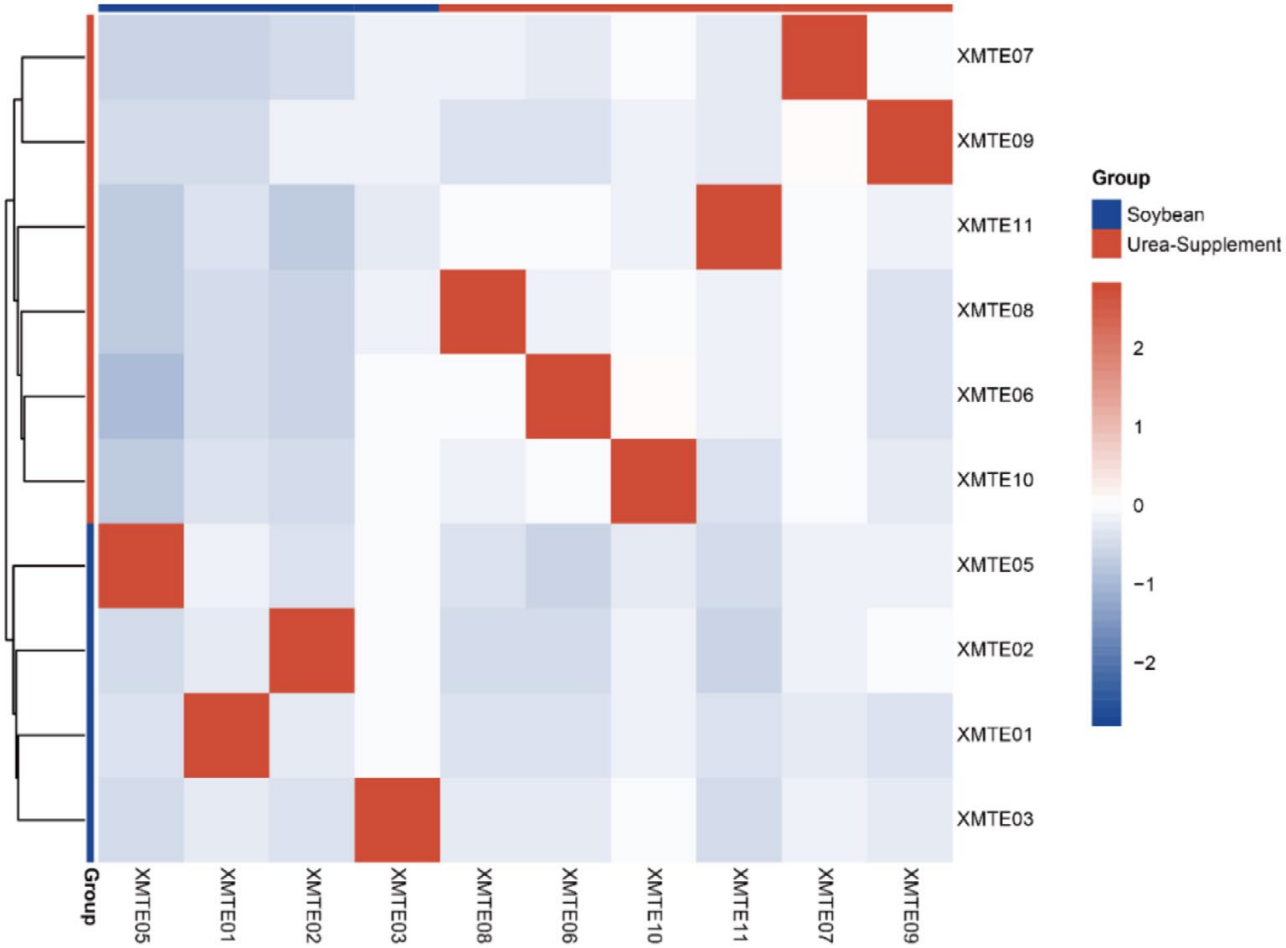
Clustering and correlation heatmap of the rumen epithelial cell transcriptome. The clustering tree on the left represents the level of similarity between samples, the up‐cluster is the urea fed group (*n* = 6) while the down‐cluster is the soybean fed group (*n* = 4). The gradient from blue to red in the correlation heatmap represents the correlation level from low to high.

Heatmaps were drawn based on the gene expression of different samples depicting the correlations and clustering relationships amongst them (Figure 2). The clustering tree on the left side of the heat map shows that the control samples fed soybean meal and the samples from the experimental group were fed with gelatinized urea instead of soybean meal, formed two main clusters, respectively. Here, the experimental group (Urea‐Supplement) (*n* = 6) showed a clustering trend, while, the four samples of the control group (Soybean) were also clustered together which is consistent with our experiment, suggesting that the treatment of urea in place of soybean meal had an effect on gene expression in the samples. In the correlation results between samples, red color indicates high correlation, while blue or white color indicates lower or no correlation respectively.

**FIGURE 3:**
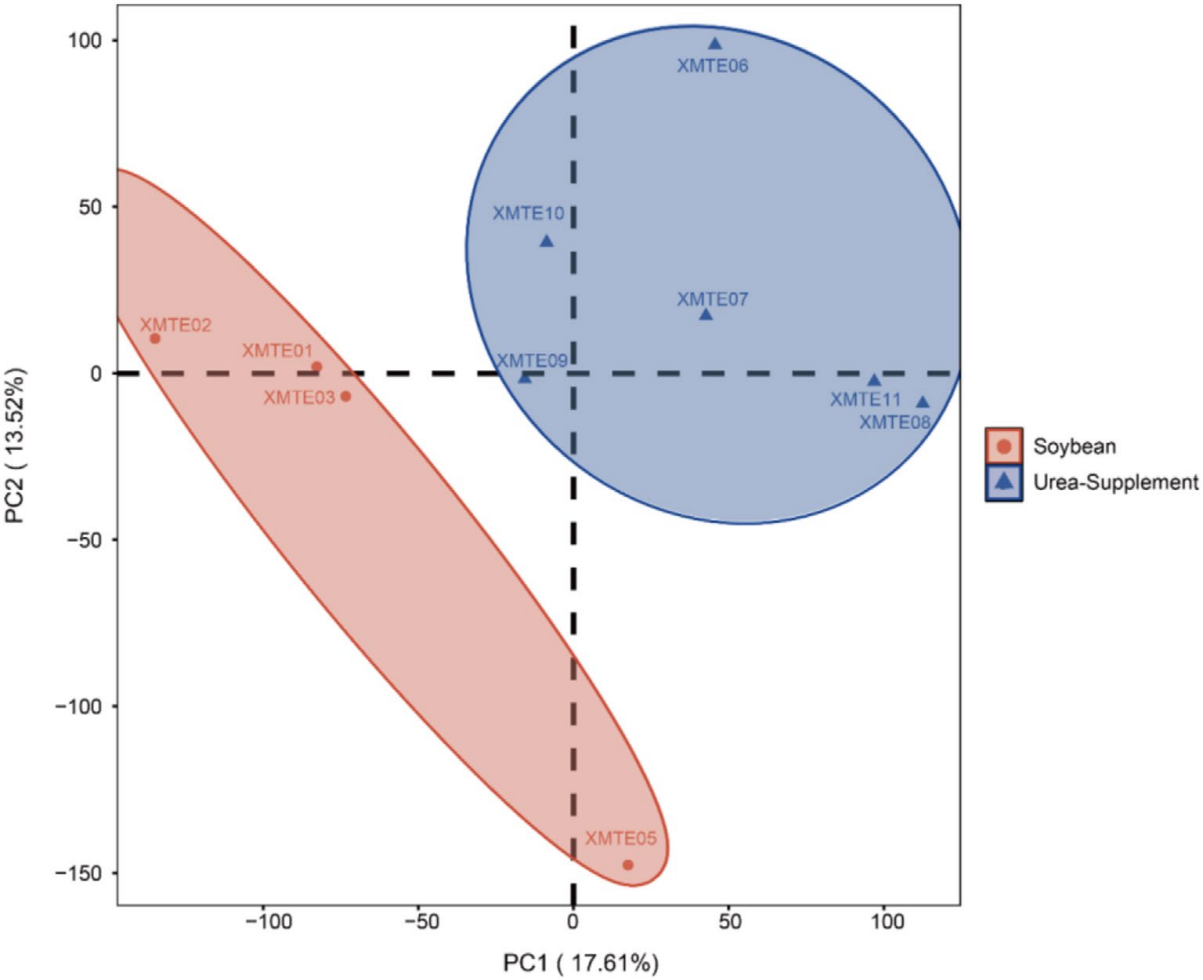
PCA plot of rumen epithelial transcriptomes from cattle in urea‐supplement and soybean meal groups. Samples from the soybean meal group (red, *n* = 4) and urea‐supplement group (blue, *n* = 6) are plotted along the first two principal component axes (PC1 and PC2).

The PCA plot shows that the gene expression profiles of the control and treatment groups belong to two different clusters (Figure 3). The gene expression profiles of the control group samples were clustered together, while those of the treatment group samples were clustered in another group. This suggests that the treatment of urea in place of soybean meal did lead to differences in the gene expression patterns of the two groups of samples.

PC1 explained 17.61% of the total variation and was the main source of variation, it can be clearly seen that there was a significant separation between the experimental and the control group on PC1, indicating that the treatment of urea supplement meal did cause changes in gene expression in the samples of the test group. However, one individual from soybean fed (XMTE05) were found in the close vicinity to each other. PC2 (second principal component) explained 13.39% of the total variation and is the secondary source of variation. The distribution of PC2 showed that the samples in the experimental group were more dispersed in the direction of PC2, while the soybean fed group was more concentrated.

### 3.3 Genes Differentially Expressed in Rumen Epithelial Cells After Dietary Shifts

**FIGURE 4:**
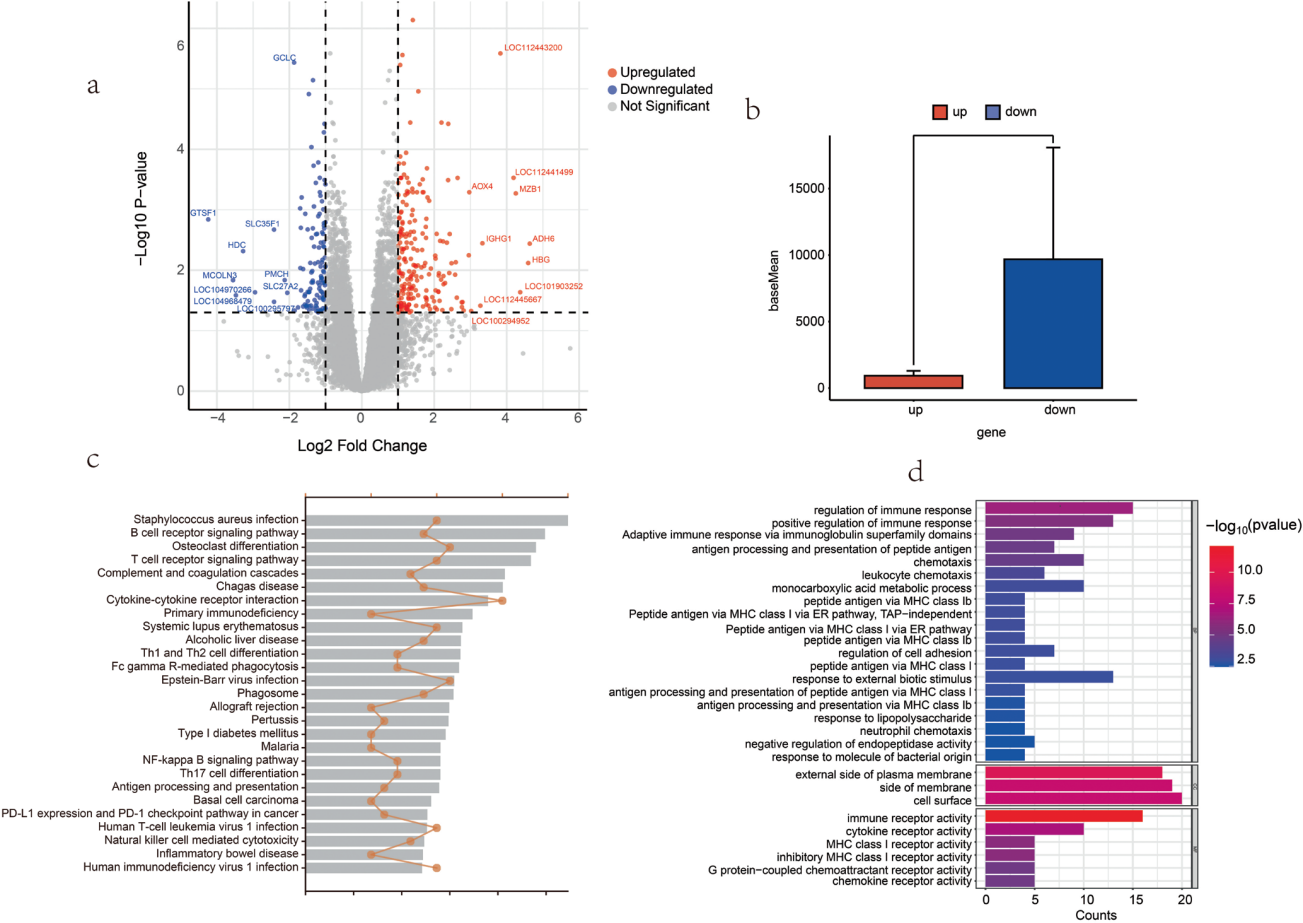
Comparative analysis of the two groups. (a) Volcano plot demonstrates the differential genes with a screening threshold of *p*‐adjusted < 0.05 and log_2_FC (fold change) > 1 or < −1, and labels the top ten genes with Log_2_FC > 2; (b) Box plot demonstrates the difference in the average expression of up‐regulated and down‐regulated gene samples; (c) Pathways that the differential genes were enriched to on KEGG; (d) Pathways enriched for differential genes on GO, the pathways retained a representative 29 BP‐related entries memorizing all CC and MF pathways, and all GO pathways are shown in the accompanying figure.

The differential gene screening threshold was *p*‐adjusted < 0.05, log_2_FC (fold change) > 1 or < −1, a total of 337 differential genes were considered as DEGs, of which 121 genes were down‐regulated and 216 genes were up‐regulated (Figure 4a) (Table S1). Despite the higher number of up‐regulated genes, the average expression of samples with down‐regulated genes was higher than that of up‐regulated genes by using the Wilcoxon way to test, and the down‐regulated genes may have more prominent roles in biological functions after the experimental treatments (Figure 4b).

GO and KEGG enrichment analyses were performed on all the DEGs. Here, KEGG enriched 27 entries, 12 of which were related to Immunity, 7 to Infectious Disease, and 8 to others (Figure 4c) (Table S2). Moreover, GO annotations revealed 90 entries of GO pathways, 81 of which were related to Biological Process, 3 to Cellular Component and 6 with Molecular Function, similar to KEGG, most of these pathways were related to immunity, inflammatory response, stress and metabolism, and MHC immune‐related pathways (Figure 4d) (Table S3).

### 3.4 The GSEA Analysis Between the Soybean and Urea‐Supplement Group

**FIGURE 5:**
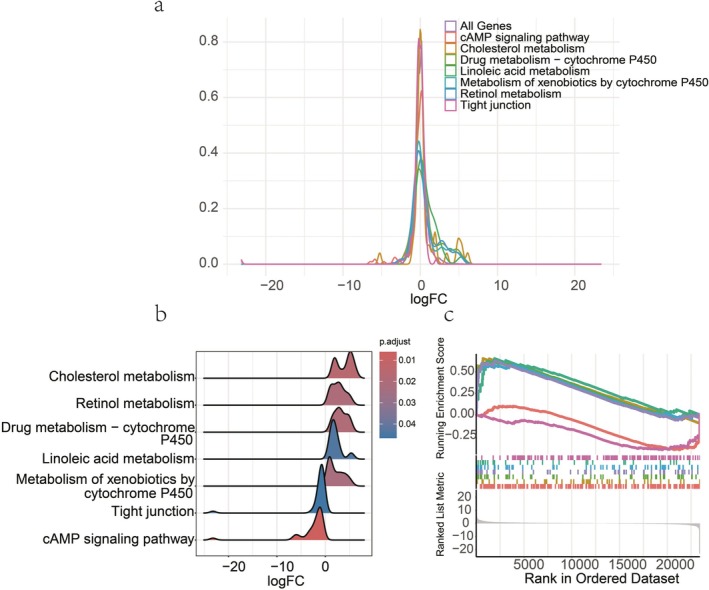
The GSEA analysis between the soybean and urea‐supplement group in KEGG. (a) A density plot shows logFC distribution of all genes in the pathway; (b) a plot showing the logFC distribution of only core_enrichment genes in the pathway related to lipid metabolism; (c) GSEA plot for the pathway related to lipid metabolism, with a down‐regulation trend for all pathways in the plot.

A total of 23 KEGG pathways were enriched using GSEA, of which 15 entries were related to immunity, and all of these pathways showed a tendency to be up‐regulated (Table 1), suggesting that the body accordingly generated an immune response in response to changes in nutrient composition in the diet, which urea was fed instead of 10% soybean meal (Figure 5).

**TABLE 1:** Biological functions and pathways significantly (*p*‐adjusted < 0.05) enriched in KEGG using GSEA.

Category	KEGG ID	Description	Number of genes	NES	*p*‐adjusted	Tendency
Metabolism‐related	bta04024	cAMP signaling pathway	220	−1.709	6.054E‐03	Down
	bta04979	Cholesterol metabolism	47	2.015	1.530E‐02	Up
	bta00982	Drug metabolism—cytochrome P450	55	1.964	1.908E‐02	Up
	bta00830	Retinol metabolism	54	1.966	2.021E‐02	Up
	bta00980	Metabolism of xenobiotics by cytochrome P450	60	1.839	2.270E‐02	Up
	bta00591	Linoleic acid metabolism	29	1.858	4.396E‐02	Up
Immune responses	bta04610	Complement and coagulation cascades	86	2.516	3.340E‐08	Up
	bta05150	*Staphylococcus aureus* infection	84	2.213	5.586E‐05	Up
	bta05171	Coronavirus disease—COVID‐19	281	1.910	1.233E‐04	Up
	bta04613	Neutrophil extracellular trap formation	126	2.040	7.550E‐04	Up
	bta05330	Allograft rejection	50	2.177	1.610E‐03	Up
	bta05332	Graft‐versus‐host disease	58	2.133	4.621E‐03	Up
	bta05322	Systemic lupus erythematosus	82	2.024	5.221E‐03	Up
	bta04612	Antigen processing and presentation	83	1.944	6.204E‐03	Up
	bta04650	Natural killer cell mediated cytotoxicity	151	1.798	6.204E‐03	Up
	bta05320	Autoimmune thyroid disease	54	2.088	6.207E‐03	Up
	bta04145	Phagosome	165	1.720	9.151E‐03	Up
	bta05340	Primary immunodeficiency	39	2.061	1.398E‐02	Up
	bta04662	B cell receptor signaling pathway	92	1.810	1.661E‐02	Up
	bta04640	Hematopoietic cell lineage	102	1.785	1.661E‐02	Up
	bta04061	Viral protein interaction with cytokine and cytokine receptor	91	1.800	2.021E‐02	Up
Other	bta04530	Tight junction	167	−1.632	4.663E‐02	Down
	bta04261	Adrenergic signaling in cardiomyocytes	146	−1.743	2.409E‐02	Down

**FIGURE 6:**
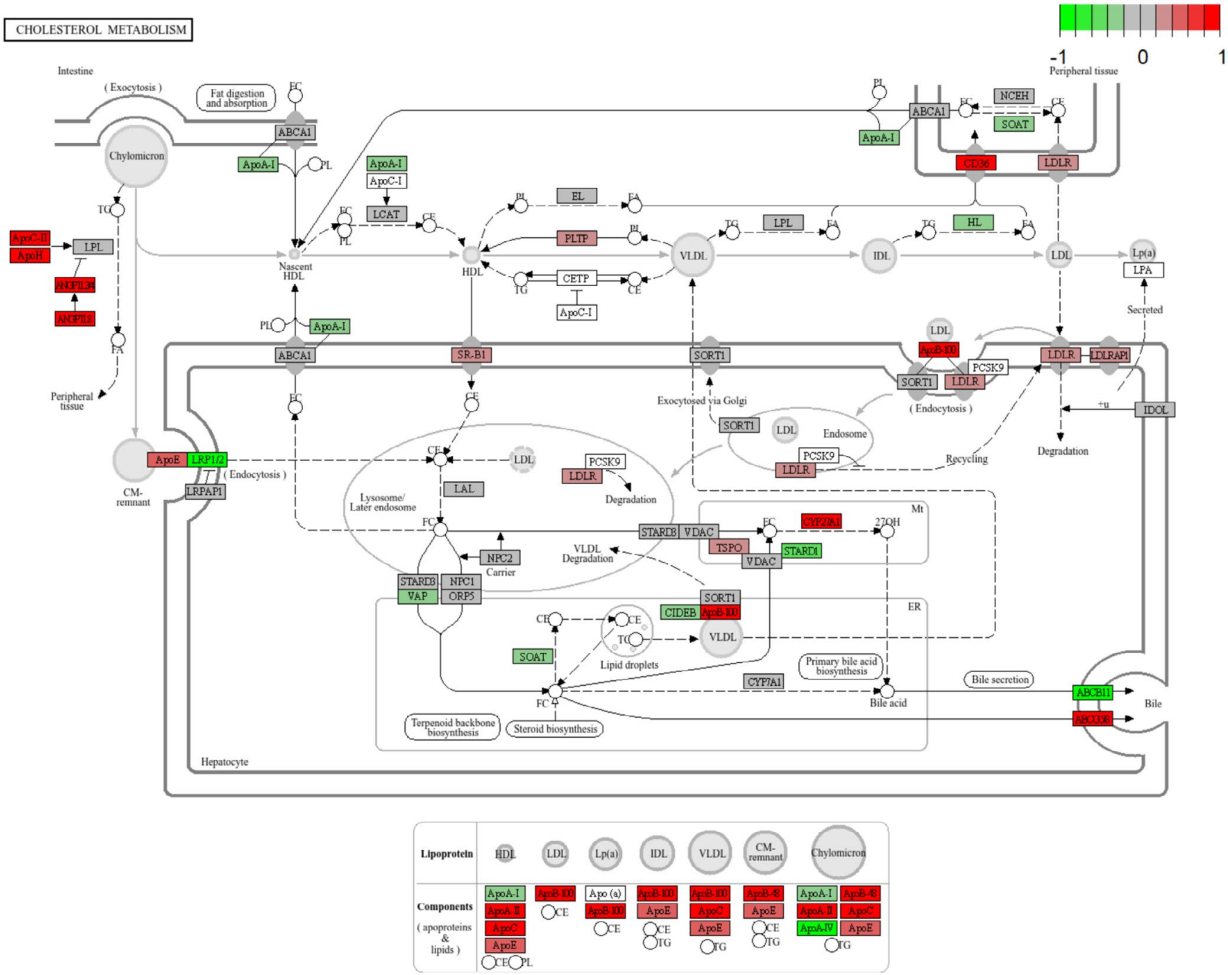
KEGG pathway for cholesterol metabolism. Genes with increased expression in urea‐supplement rumen epithelial cells are shown in red, and down‐regulated genes are shown in green.

**FIGURE 7:**
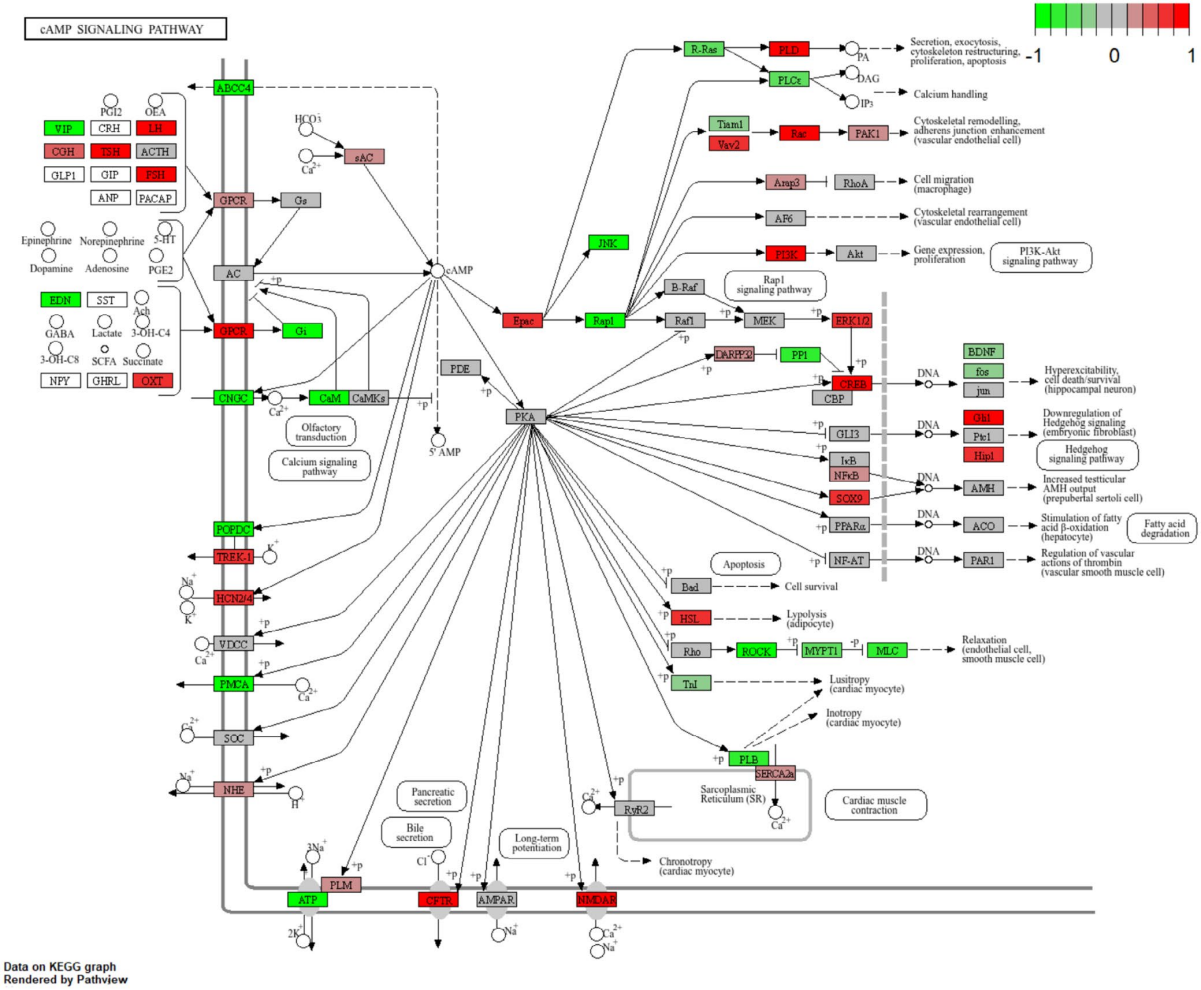
KEGG pathway of cAMP signaling pathway. Genes with increased expression in urea‐supplement rumen epithelial cells are shown in red, and down‐regulated genes are shown in green.

**FIGURE 8:**
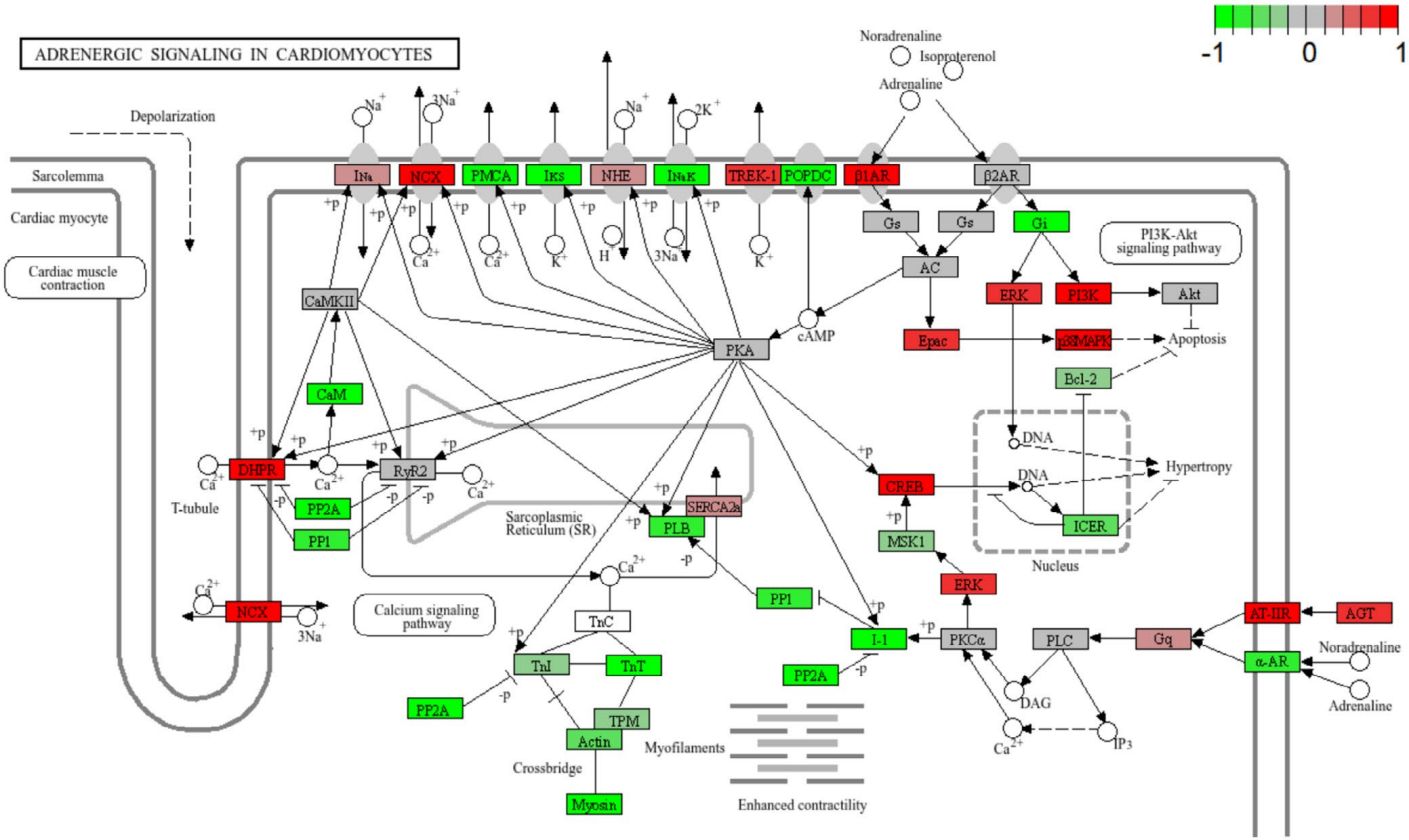
KEGG pathway of adrenergic signaling in cardiomyocytes. Genes with increased expression in urea‐supplement rumen epithelial cells are shown in red, and down‐regulated genes are shown in green.

**FIGURE 9:**
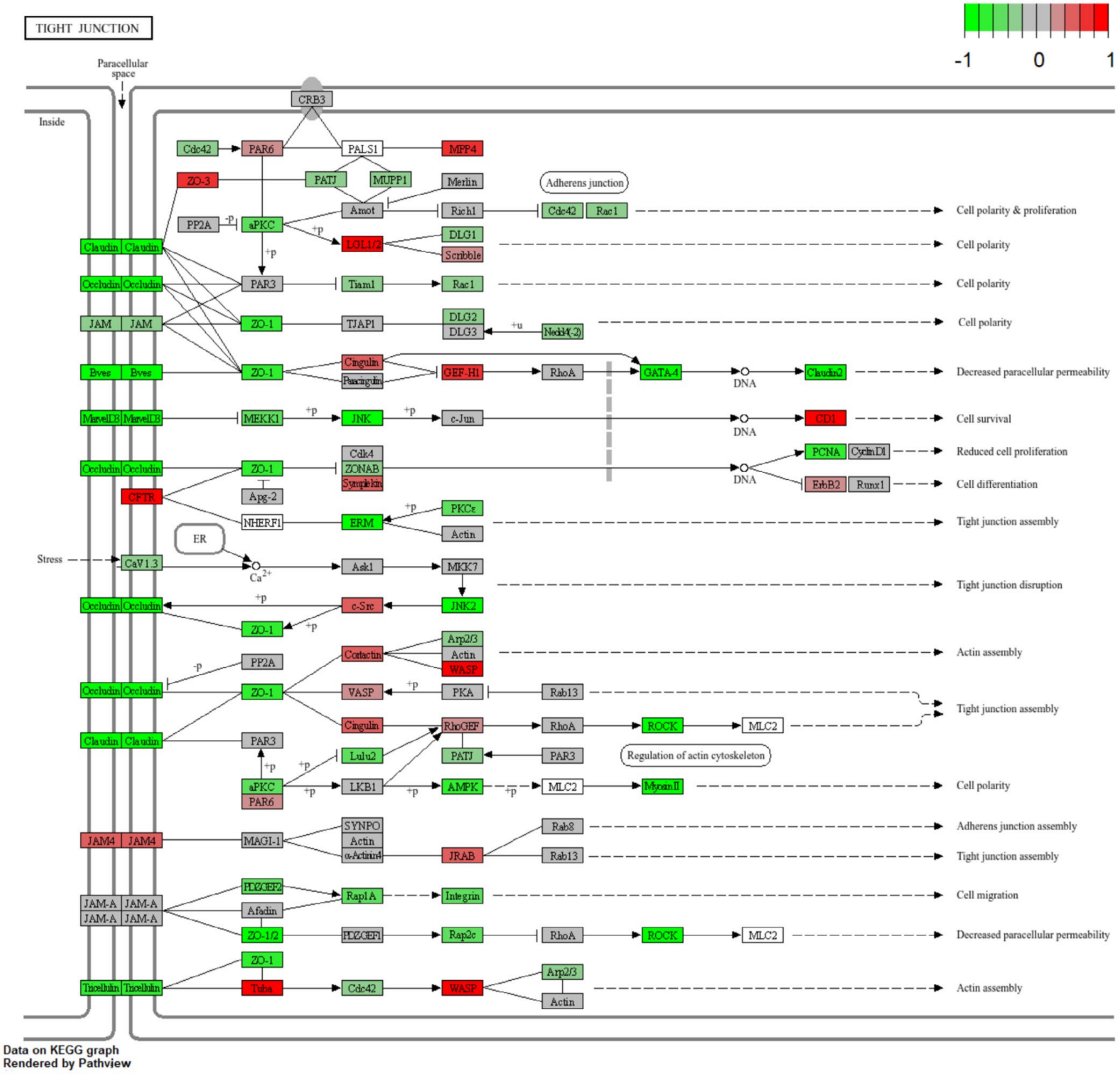
KEGG pathway of tight junction. Genes with increased expression in urea‐supplement rumen epithelial cells are shown in red, and down‐regulated genes are shown in green.

The complete regulatory mechanism of the cholesterol metabolism pathway can be seen in the fact that the components that make up the various types of lipoproteins, represented up‐regulation (Figure 6) (Hao et al. 2025). However, cAMP signaling pathway (bta04024) did not show up‐regulation trend playing an important role in regulating lipid metabolism and hormone signaling. Furthermore, the complete presentation of this KEGG pathway showed the upregulation of HSL gene, which is synthesized with lypolysis (adipocyte) (Figure 7). Besides, adrenergic signaling in cardiomyocytes (bta04261) represented down‐regulation, playing a critical role in regulating the contractility and heart rate of cardiomyocytes. This pathway typically operates through β‐adrenergic receptors (β1AR and β2AR), which mediate cAMP signaling and calcium (Ca^2+^) signaling to control cardiomyocyte function (Sellers et al. 2009; Haodi et al. 2014; Department of Physiology and Biophysics, and Mississippi Center for Obesity Research 2019; Eliezeck et al. 2024). Under the condition of replacing 10% of soybean meal with urea, cardiomyocytes appear to reduce the sensitivity of calcium signaling and adrenergic signaling, thereby lessening the cardiac load. This may serve as a protective response to adapt to the metabolic stress induced by changes in nitrogen sources (Figure 8). In addition, a pathway that deserve attention is the Tight junction (bta04530), which is associated with the maintenance of cellular connectivity and the establishment of barriers, and down‐regulation of this pathway represented increased cell permeability, the decrease in the tightness of urea, a hydrophilic substance, also accelerates the absorption of nutrients from the rumen epithelium (Figure 9).

**FIGURE 10:**
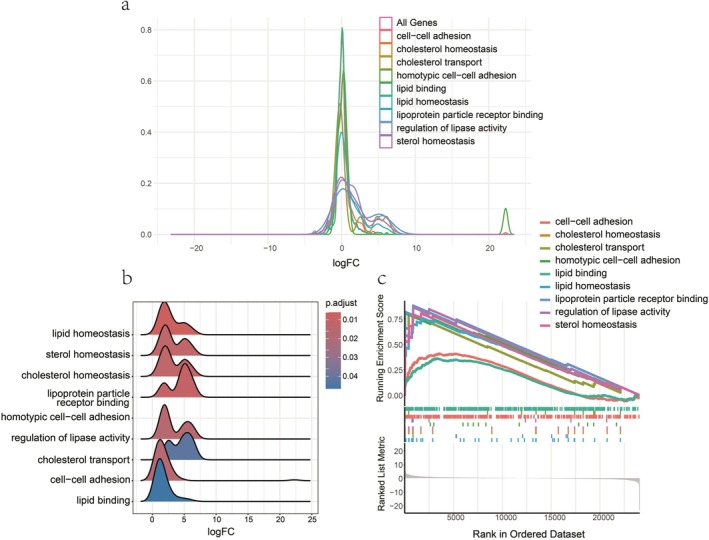
The GSEA analysis between the soybean and urea‐supplement group in GO: (a) A density plot shows logFC distribution of all genes in the pathway; (b) A plot showing the logFC distribution of only core enrichment genes in the pathway related to lipid metabolism; (c) GSEA plot for the pathway related to lipid metabolism, with a down‐regulation trend for all pathways in the plot.

GSEA enriched a total of 153 GO pathways, all related to immunity, lipid metabolism and substance transport, and internal environment homeostasis (Table S4). The same results as KEGG analysis were associated with immunity, lipid metabolism (GO:0055088: lipid homeostasis, GO:0070325: lipoprotein particle receptor binding, GO:0042632: cholesterol homeostasis, GO:0055092: sterol homeostasis, GO:0060191: regulation of lipase activity, GO:0030301: cholesterol transport, and GO:0008289: lipid binding) pathways were all shown to be up‐regulated in the urea replacement group. Moreover, the cell adhesion interactions pathway showed upregulation (GO:0098609: homotypic cell–cell adhesion and GO:0034109: cell–cell adhesion) (Figure 10).

### 3.5 The Analysis of Phenotypic Differences

Based on the measuring data, no significant differences were observed between the two treatment groups of Simmental bulls in initial weight, final weight, total weight gain, or average daily gain (*p* > 0.05) (Table S6). Rumen pH, ammonia nitrogen, acetate, propionate, isobutyrate, butyrate, and total volatile fatty acid concentrations showed no significant changes between these two groups (*p* > 0.05) (Table S6).

## 4 Discussion

The use of urea‐supplement meal instead of all soybean meal can greatly reduce the feed cost, but while reducing the feed cost, the animal well‐being is on the edge. Addressing the welfare and even productive improvement of the animal is the need of the hour (Wang 2003; Highstreet et al. 2010; Spanghero et al. 2018). In this study, RNA‐seq was performed on the rumen epithelial cells after feeding urea to replace 10% soybean meal, and analyzes genes that were differentially expressed in the urea and the soybean meal group.

The heatmap and PCA plot revealed that gene expression patterns were more similar within each group but showed clear differentiation between the experimental and control groups. This suggests that the treatment of urea replacing 10% soybean meal did have a certain degree of influence on gene expression. There was a certain degree of heterogeneity in the gene expression response of some individuals within the group, which may suggest that there are differences in the sensitivity of different individuals to the treatments.

Nearly two‐thirds of the genes in 337 differentially expressed genes were up‐regulated, whereas the average expression of the down‐regulated genes was significantly higher than that of the up‐regulated genes, and when the down‐regulated genes were enriched, they were not enriched for any significant pathways. However, traditional KEGG and GO pathway analysis of all differential genes enriched for immune‐regulatory, inflammatory response, stress, and metabolism‐related pathways, suggesting that these pathways may be affected by feed alterations and might tend to be up‐regulated.

To test this conjecture, we used GSEA enrichment analysis to assess whether the cumulative trend was up or down tendency after feeding changes. GSEA analysis enriched more than half of the KEGG immune pathways, and all of these pathways tended to be up‐regulated, suggesting that the body has a stress response due to changes in the diet, activating the body's immune system to maintain homeostasis in the internal environment. The cytochrome P450 (CYP450) system is an important detoxification enzyme system, which plays a key role in the metabolism of exogenous substances, drugs and endogenous metabolites. Here, the upregulation of bta00982 and bta00980 related to the metabolism of exogenous substances by cytochrome P450, suggested that the organism increased detoxification metabolism in response to changes in dietary composition, in order to break down or eliminate potentially toxic by‐products (Guo et al. 2024; Miksys et al. 2024; Chen et al. 2025). Additionally, pathways involved in cholesterol metabolism (bta04979), retinol metabolism (bta00830), and linoleic acid metabolism (bta00591) were upregulated. These pathways have important roles in regulating cell membrane structure, hormone synthesis and antioxidant defense. The up‐regulation trend may indicate enhanced lipid and vitamin metabolism to maintain energy supply and antioxidant homeostasis when adapting to a new nitrogen source (urea), suggesting that lipid metabolism can respond to changes in dietary composition. The complete regulatory mechanism of the cholesterol metabolism pathway can be seen in the fact that the components that make up the various types of lipoproteins, represented up‐regulation (Figure 6) (Hao et al. 2025).

Cholesterol plays an important role in the meat quality of beef cattle (Sahin et al. 2021; Aksoy et al. 2021), and in our results, the pathway related to cholesterol metabolism showed an up‐regulation trend, and to understand the complete mechanism of this pathway. The up‐regulated genes are related to the synthesis of various types of lipoproteins, suggesting that in the situation of a decrease in the intake of lipids triggers the rumen epithelium to take measures to synthesize lipoproteins (Kaizhi et al. 2023), to maintain the homeostatic state of the body's internal lipids. The current study also proved that there was no significant difference in meat quality was observed between the two groups of cattle after feeding urea instead of 10% soybean meal, which indicated that feeding moderate urea to replace a part of soybean meal did not have adverse effects on the meat quality of beef cattle while reducing the cost of feed (Zhang et al. 1998; Sun 2009; Deng et al. 2013). Genes related to the synthesis of adipocytes in the cAMP signaling pathway were also shown to be significantly up‐regulated, which further supported the existence of this compensatory mechanism.

Xin et al. studied the regulatory mechanisms of Tight Junctions in the intestinal epithelium and found that a stronger barrier is not always better. Some less toxic hydrophilic drugs, for example, cannot be absorbed through the transcellular pathway when the barrier is too tight, which affects their bioavailability (Georgia and Germany et al. 2005; Ma et al. 2005; Lorenza et al. 2008; Hee 2015; Cong et al. 2016). Further investigation into the adherens junction pathway (Kong et al. 2016) revealed that the rumen epithelium is highly metabolically active due to its high mitochondrial density, particularly in the stratum basale layer (Graham and Simmons 2005). This metabolic activity enables the rumen epithelium to process a significant proportion of absorbed short‐chain fatty acids (SCFAs) (Sehested et al. 1999). They made a detailed study on the absorptive capacity of rumen epithelial cells by linking phenotypic data and metabolomics, which down‐regulated the adhesion of rumen epithelial cells in its L‐RFI animals, leading to larger cell gaps and increased permeability, which made the epithelial cells exposed to nutrients at a faster rate, which was conducive to increase the nutrient absorptive capacity of rumen epithelial cells. In our study, the observed downregulation of the Tight Junction pathway following the replacement of 10% soybean meal with urea suggests a similar trend. Under conditions of lower nutrient supply, reduced intercellular adhesion and increased permeability may reflect structural changes in the rumen epithelium or an expansion of the absorption area. This, in turn, could be a compensatory mechanism that enhances nutrient uptake efficiency in response to the altered diet (Bruewer et al. 2005; Ma et al. 2005; Basuroy et al. 2006; Hee 2015).

In addition, the up‐regulation of intercellular interactions in the GO pathway reflects that the organism may have adopted dynamic regulation to prevent ammonia uptake into the bloodstream and to ensure the stability of the rumen environment under urea‐substituted soybean meal feeding. This molecular response is highly consistent with our physiological outcomes, as feeding Simmental bulls with diets in which 0.75% gelatinized urea replaced part of the soybean meal resulted in no notable differences in growth performance, rumen fermentation parameters, or ammonia nitrogen concentrations.

## 5 Conclusion

The findings of this study suggest that urea replacement of some soybean meal feeding activates compensatory mechanisms in the rumen epithelium to maintain homeostasis of the organism through dynamic changes in intercellular junctions and adaptive up‐regulation of metabolic pathways, which is scientifically consistent with a reasonable expectation of a metabolic stress response induced by feed substitution. Moreover, replacement of dietary nitrogen sources may potentially enhance nutrient absorption and immune capacity in the long term; however, the extent and stability of intercellular junctional changes require further investigation. Therefore, additional studies integrating phenotypic data and metabolic outcomes are warranted to ensure the long‐term safety and efficacy of this feeding strategy.

## Author Contributions

**Yushan Li:** formal analysis (lead), investigation (lead), methodology (lead), software (equal), visualization (lead), writing – original draft (lead), writing – review and editing (equal). **Xinyi Li:** formal analysis (supporting), investigation (supporting), methodology (supporting), validation (supporting), visualization (supporting), writing – original draft (supporting), writing – review and editing (equal). **Xinxin Sui:** data curation (equal), investigation (supporting), methodology (equal), validation (equal), writing – review and editing (equal). **Li Liu:** data curation (equal), funding acquisition (supporting), resources (equal), validation (equal), writing – review and editing (equal). **Quratulain Hanif:** conceptualization (supporting), methodology (supporting), writing – original draft (supporting). **Fuwen Wang:** formal analysis (supporting), methodology (supporting), software (supporting). **Li Luo:** data curation (supporting), methodology (supporting), validation (supporting). **Ye Bu:** data curation (supporting), software (supporting), validation (supporting). **Shanshan Xu:** data curation (supporting), validation (supporting). **Yue Wang:** data curation (supporting), software (supporting). **Chuzhao Lei:** conceptualization (equal), project administration (equal), supervision (lead). **Fang Sun:** data curation (lead), project administration (equal), resources (lead).

## Ethics Statement

The authors confirm that the ethical polices of the journal, as noted on the journal's author guidelines page, have been adhered to. Also, the cattle participating in the study were managed and cared for in accordance with the protocol approved by the Science and Technology Ethics Committee of Heilongjiang Bayi Agricultural University, and the approved protocol number for this study was DWKJXY2024006.

## Conflicts of Interest

The authors declare no conflicts of interest.

## Supporting information


**Table S1:** The DEGs between the Soybean group and the Urea group.
**Table S2:** KEGG results from the enrichment analysis of DEGs.
**Table S3:** GO results from the enrichment analysis of DEGs.
**Table S4:** KEGG and GO results from the enrichment analysis of GSEA.
**Table S5:** Composition and nutrient levels of experimental diets (DM basis) %.
**Table S6:** Performance of Linear Growth in Simmental Bulls Before and After Feeding.

## Data Availability

The data utilized in the manuscript has been uploaded to the NCBI. The project ID for this data is PRJNA1241987.
